# Glial Fibrillary Acidic Protein (GFAP)-Expressing Metastatic Carcinoma to the Brain: A Case Report

**DOI:** 10.7759/cureus.107839

**Published:** 2026-04-27

**Authors:** Benjamin L Wang, Taylor G Brown, Darby BeDell, Stuart E Cameron, Liam L Chen

**Affiliations:** 1 Laboratory Medicine and Pathology, University of Minnesota Medical School, Minneapolis, USA; 2 Laboratory Medicine and Pathology, Hennepin County Medical Center, Minneapolis, USA

**Keywords:** brain metastasis, dicer1 mutation, glial fibrillary acidic protein, glioblastoma, lung adenocarcinoma

## Abstract

Presented here is a case of a 69-year-old man with undefined lung nodules who was found to have enhancing masses in the right superior frontal and right precentral gyri. The tumor displayed pathologic features initially suggestive of glioblastoma, including diffuse but atypical glial fibrillary acidic protein (GFAP) expression, positive but weak oligodendrocyte transcription factor 2 (Olig2) staining, high mitotic activity, and pseudopalisading necrosis. However, next-generation sequencing revealed no glioblastoma-associated genetic alterations but did reveal a mutation in *DICER1*, a gene encoding an RNA endoribonuclease implicated in malignant lung tumors. Further investigation via tumor methylation profiling revealed a strong match with lung adenocarcinoma. This case offers a rare presentation of GFAP-positive lung adenocarcinoma metastasis to the brain and validates the combined histological, immunohistochemical, and molecular approach to brain tumor diagnosis.

## Introduction

Metastases are the most common cause of brain tumors and a complication in 10%-40% of patients with metastatic cancer [1,2]. The majority of tumors originate from primary cancer of the lungs (>50%), breasts (15%-25%), or skin (5%-20%), and the vast majority of metastatic brain tumors localize to the cerebrum (80%) or the cerebellum (15%) [3]. On the other hand, primary brain tumors may be benign or malignant, with more than 70% of tumors being glial in origin [3] and more than 50% of these gliomas being classified as glioblastomas (WHO grade 4) with extremely poor prognosis [4].

Recent glioblastoma therapy research has created exciting alternatives and treatment strategies complementing surgical resection and radiotherapy, such as targeted p53-regulator MDM2 proto-oncogene inhibitors [5], checkpoint inhibitors to weaken glioblastoma-specific tumor immunosuppression [6], and nanoparticles for targeted drug delivery through the blood-brain barrier [7]. However, the targeted nature of these therapies necessitates accurate diagnosis of glioblastoma from other metastatic tumors. To complicate matters, clinical presentation of metastatic and primary brain tumors has high similarity, with both patient populations commonly experiencing intracranial hypertension, seizures, headache, and/or focal neurologic symptoms [1,3]. Therefore, accurate differentiation between primary and metastatic tumors is a priority in effective management of brain tumors.

Presented here is a case of a 69-year-old man found to have right frontal tumors displaying unique pathologic features. Initial histological examination and immunohistochemical analyses revealed characteristics highly suggestive of high-grade glioma, yet genetic profiling was strongly indicative of metastatic carcinoma. This case highlights the variability of cancer tumor characteristics and the advantages in genetic profiling for tumor diagnosis and subsequent treatment.

## Case presentation

Clinical history

A 69-year-old male with a past medical history of pulmonary nodules, epilepsy, chronic obstructive pulmonary disease, hypertension, cardiomyopathy, coronary artery disease, latent tuberculosis infection, alcohol-use disorder, and tobacco-use disorder presented to the emergency department with confusion and left hemiparesis, causing a series of falls over the previous two days. He was noted to have normal pulmonary effort but appeared cachectic. On exam, shoulder abduction, elbow flexion and extension, wrist flexion and extension, hand grip, hip flexion, knee flexion, dorsiflexion, and plantar flexion were significantly weaker on the left than the right.

The patient had multiple known pulmonary nodules including a biopsy-proven granulomatous left upper lobe nodule, a calcified solid 1.4 cm right lower lobe nodule, and several punctate nodules in the right upper lobe. Biopsies taken from the left upper lobe nodule six months prior to the onset of neurologic symptoms were positive for necrotizing granulomatous inflammation but negative for mycobacteria or fungus. Samples taken via fine needle aspiration were negative for malignancy at the time. The patient also had three lymph nodes sampled concurrently with the biopsy which did not demonstrate signs of malignancy.

Radiology

**Figure 1:**
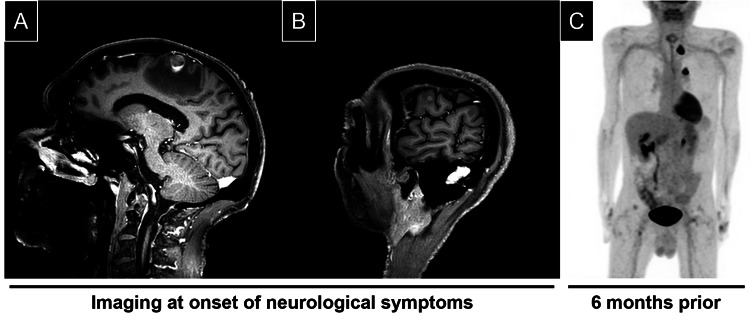
Brain lesions identified in patient with history of non-malignant lung nodules Magnetic resonance imaging (MRI) of brain lesions with significant surrounding edema (A-B). Lesions identified in the right superior frontal gyrus measuring 1.5 x 1.2 x 1.3 cm (A) and in the right precentral gyrus measuring 0.5 x 0.5 x 0.5 cm (B). Long-axial positron emission tomography (PET) scan six months prior demonstrated high physiological uptake in lung nodules, which was interpreted as non-malignant (C).

A computed tomography (CT) head scan was negative for intracranial hemorrhage but discovered a right frontal subcortical confluent hypodensity with local mass effect, concerning for a possible metastatic lesion. Brain magnetic resonance imaging (MRI) revealed peripherally enhancing lesions in the right superior frontal gyrus (1.5 x 1.2 x 1.3 cm) and in the right precentral gyrus (0.5 x 0.5 x 0.5 cm) (Figures 1A, 1B). There was significant surrounding edema around both lesions. These lesions were not present six months prior, when the patient received a long-axial positron emission tomography with fluorodeoxyglucose F18 tracer (18F-FDG PET) scan for the lung nodules (Figure 1C).

Surgery

The patient underwent a bicoronal craniotomy for resection of the right frontal tumor. Phase reversal was used intraoperatively to identify the primary motor cortex. The tumor was located in the premotor cortex.

Post-Surgical Course

In the postoperative period, the patient had increased left hemiparesis, likely due to the proximity of the tumor to the supplemental motor area. He slowly improved through his hospital course to near preoperative deficit levels and was discharged to a subacute rehabilitation facility. He experienced persistent left hemiparesis but had reservations about chemotherapy and radiation therapy. Ultimately, he opted to pursue hospice care and died after one month in hospice.

Pathology

Gross

A biopsy specimen was obtained from the right frontal brain lesion for intraoperative consultation consisting of a 0.6 x 0.6 x 0.5 cm disrupted pink to red colored, slightly mucinous, with soft, spongy consistency tissue fragment. The frozen section remnant was entirely submitted for permanent section. Additionally, a 1.3 x 1.0 x 0.5 cm aggregate of focally disrupted, dusky tan-pink friable soft tissue fragments was obtained and entirely submitted for permanent section.

Microscopic

**Figure 2:**
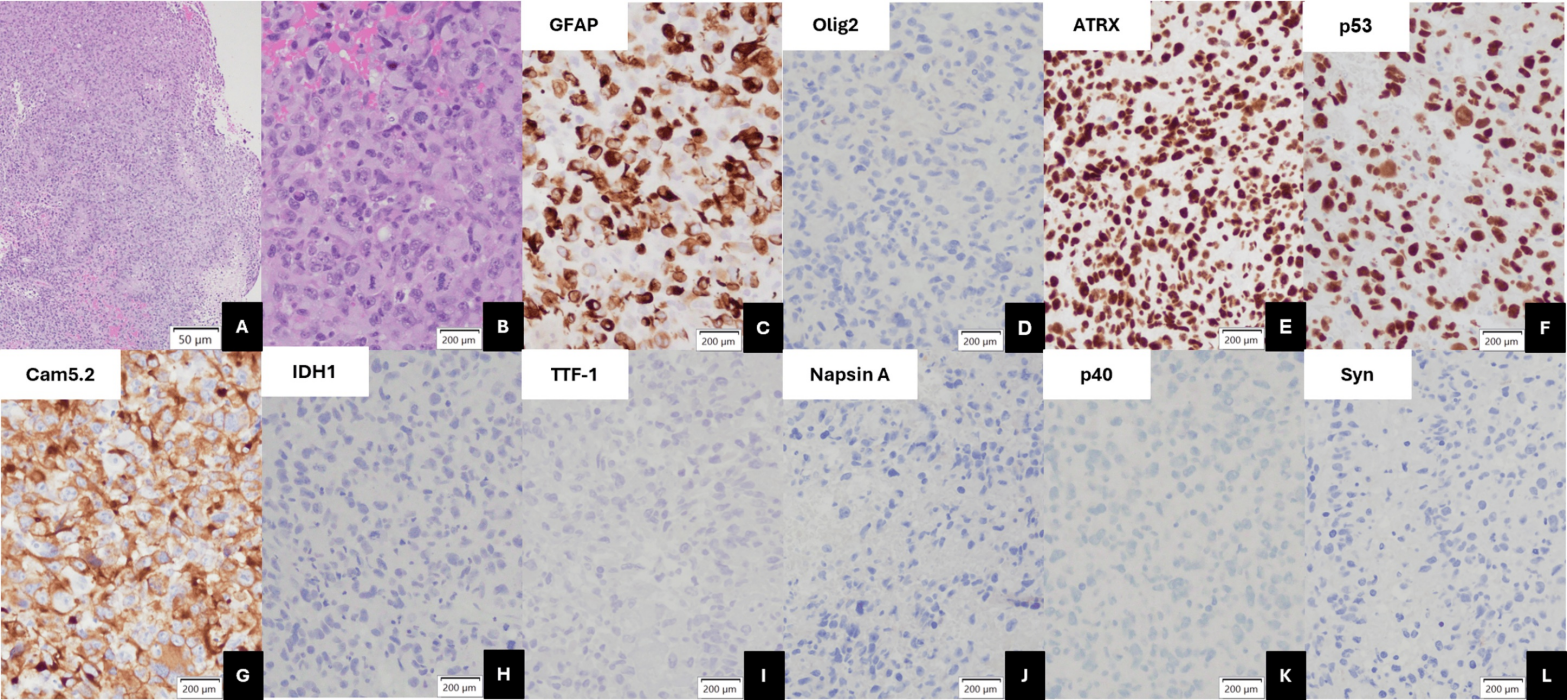
Unusual immunohistochemical marker expression in tumor biopsy Hematoxylin and eosin stain demonstrating tumor pseudopalisading necrosis (A; 4x) and brisk mitotic activity (B; 20×). Immunohistochemical staining of the tissue assayed for (C) GFAP; (D) Olig2; (E) ATRX; (F) p53; (G) Cam5.2; (H) IDH1-R132H variant; (I) TTF-1; (J) Napsin A; (K) p40; and (L) Syn reactivity. All immunohistochemical images were taken at 20× power. GFAP, glial fibrillary acidic protein; Olig2, oligodendrocyte transcription factor 2; ATRX, alpha-thalassemia mental retardation X-linked; IDH1, isocitrate dehydrogenase 1; TTF-1, thyroid transcription factor 1; Syn, synaptophysin.

Hematoxylin and eosin (H&E)-stained sections showed tissue with brisk mitotic activity and pseudopalisading necrosis with no definitive microvascular proliferation (Figures 2A, 2B). The tumor cells demonstrated diffuse and strong positive labeling for astrocyte marker GFAP (Figure 2C) and positive but weak oligodendrocyte marker oligodendrocyte transcription factor 2 (Olig2) (Figure 2D). The tumor also had retained transcriptional regulator alpha-thalassemia mental retardation X-linked (ATRX) labeling (Figure 2E), strong and diffuse p53 expression in most nuclei (Figure 2F), and the Ki-67 proliferation index was significantly elevated at 40%-50% (not shown). The tumor cells were also positive for epithelial marker Cam5.2 (Figure 2G). The tumor was negative for isocitrate dehydrogenase 1 (IDH1)-R132H variant, thyroid transcription factor 1 (TTF-1), Napsin A, p40, and synaptophysin (Figures 2H-2L).

Next-Generation Sequencing

**Figure 3:**
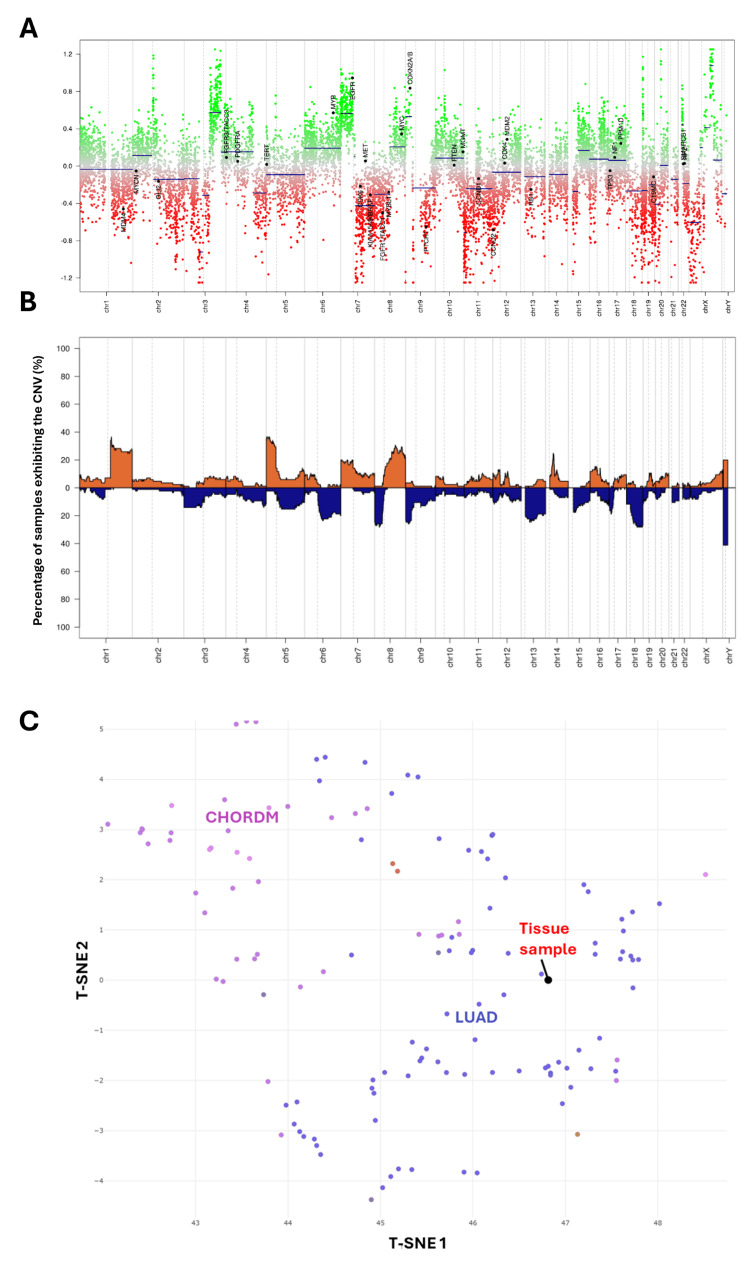
Molecular profiling supports lung adenocarcinoma origin Analysis for copy number variation analysis depicting chromosomes 1-22, X, and Y (A). Gain or amplification represented by positive variation from baseline, whereas loss represented by negative deviations from baseline. Summary copy number profile of matched class (lung adenocarcinoma, LUAD; B). t-SNE plot of tumor methylation profiles of tissue sample (C) reveals match for LUAD. Additional chordoma (CHORDM) cluster labeled shows clear distinction from LUAD and tissue sample. t-SNE, t-distributed Stochastic Neighbor Embedding; CNV, copy number variant; MDM4, MDM4 regulator of p53; MYCN, MYCN proto-oncogene, bHLH transcription factor; GLI2, GLI family zinc finger 2; FGFR3/TACC3, fibroblast growth factor 3/transforming acidic coiled-coil containing protein 3; PDGFRA, platelet-derived growth factor receptor alpha; TERT, telomerase reverse transcriptase; MYB, MYB proto-oncogene, transcription factor; EGFR, epidermal growth factor receptor; CDK, cyclin-dependent kinase; MET, MET proto-oncogene, receptor tyrosine kinase; BRAF, B-Raf proto-oncogene, serine/threonine kinase; PTCH, patched; PTEN, phosphatase and tensin homolog; MGMT, O-6-methylguanine-DNA methyltransferase; CCND, cyclin D1; RB1, RB transcriptional corepressor 1; TP53, tumor protein p53; NF1, neurofibromin 1; PPM1D, protein phosphatase, Mg2+/Mn2+ dependent 1D; C19MC, chromosome 19 microRNA cluster; SMARCB1, SWI/SNF-related BAF chromatin remodeling complex subunit B1.

B-Raf proto-oncogene, serine/threonine kinase (BRAF) G469A variant, tumor protein p53 (TP53) G245S variant, neurofibromin 2 (NF2) E408fs variant, CDKN2A c.152_457+418del, and DICER1 E497* variants were detected. Copy number analysis revealed amplifications in chromosome regions 3q25.1-3q26.31 and 20q11.21-20q11.21; shallow losses were noted in 9q13-9q33.1 and 11p15.5-11p11.12 (Figure 3A). Copy number profile summary was provided in Figure 3B. The number of somatic mutations in the tumor genome, or tumor mutational burden (TMB), for this sample was >19 mut/MB. A microsatellite stable (MSS) phenotype was detected, suggesting stable microsatellite repeat lengths similar to normal tissue.

Methylation Profile

Tissue samples were submitted for methylation profiling to the Clinical Methylation Unit in the Laboratory of Pathology at the National Cancer Institute (NCI). The tumor was negative for MGMT promoter methylation. The tissue biopsy DNA methylation profile was analyzed and compared to the NCI/Bethesda CNS Tumor classifier v2.0, and a t-distributed Stochastic Neighbor Embedding (t-SNE) plot was provided for dimensionality reduction of expression datasets (Figure 3C). The tissue biopsy methylation profile strongly matched the cluster corresponding to lung adenocarcinoma methylation with 0.99 classifier confidence score. Meanwhile, the submitted tissue exhibited no clustering overlap with glioblastoma or tumors of DICER1 gene mutation.

## Discussion

This is quite an unusual tumor presentation with diffuse and strong GFAP expression, brisk mitotic activity, and pseudopalisading necrosis. Although many of these features are suggestive of an IDH-wildtype glioblastoma, inconsistencies such as multifocal enhancing lesions, weak OLIG2 but strong CAM5.2 expressions, and most importantly, a lack of glioblastoma-associated genetic alterations do not support this diagnosis. Furthermore, methylation profiling ultimately matched the tumor to a metastatic neoplasm.

This patient had lung nodules suggestive of pulmonary malignancy, although no pathological diagnosis was established, and thus, there was a high suspicion for metastasis from the lung to the brain. There was a very rare neurofilament inside the tumor (not shown), which favored brain metastasis over a primary brain tumor. Lung adenocarcinomas have a variety of histologic subtypes and may present with glandular differentiation, mucin production, and pneumocyte marker expression [8]. They are often TTF1-positive, Napsin A-positive, and p40-negative [8,9]. This tumor was negative for all these markers. While GFAP-positive reactive astrocytes have been reported to infiltrate brain metastases [10], the atypical, polymorphic tumor cells themselves were GFAP-positive in this case. A rare instance of lung carcinoid with GFAP immunoreactivity was previously reported in 1993, although the pattern of GFAP reactivity appeared thin and filamentous [11], contrasting with the staining pattern in the present patient’s tissue biopsy. Animal studies have shown GFAP-expressing non-myelinating Schwann cells associated with pulmonary nerves in the mouse lung [12], and GFAP-associated dedifferentiated Schwann cells have been identified in human lung cancers [13]. However, to our knowledge, the occurrence of lung carcinoma expressing GFAP metastasizing to the brain has never been reported.

The tissue biopsy was found to have mutations in DICER1, a gene encoding Dicer RNA endonuclease, in which mutations have been implicated in inherited cancer DICER1 syndrome [14]. One of the most common neoplasms in this syndrome is pleuropulmonary blastoma (PPB), a rare malignant lung tumor that typically occurs in patients under age 4 [15]. Pathologically, PPB is characterized by sheets of mesenchymal cells that may appear spindle-shaped with thin-walled cysts, although there are some histological variations among subtypes [16]. Given the patient's DICER1 mutation, pleuropulmonary blastoma (PPB) metastasis to the brain was considered. Primary intracranial sarcoma is also associated with DICER1 somatic and germline mutations [4], and concurrent adult intracranial sarcomas with DICER1-mutations have been reported on rare occasions [17,18]. Intracranial sarcoma often presents histologically similarly to PPB, with prominent spindle cells with fascicular growth, brisk mitotic activity, and desmin positivity [4]. In the present patient's case, the tissue biopsy was negative for desmin and histological examination did not reveal a spindle-cell appearance, making PPB and intracranial sarcoma unlikely. In addition, DICER1 syndrome may present with central nervous system manifestations such as pineoblastoma and pituitary blastoma [14,16,17]. However, both these neoplasms contain diffuse expression of synaptophysin (not shown), which was negative in this patient’s case. Given the patient’s age and a relatively low variant allele frequency (37%), DICER1 germline syndrome was highly unlikely.

## Conclusions

In this case, multiple conflicting standard diagnostic markers appeared, such as negative TTF1 and Napsin A staining suggesting non-lung adenocarcinoma (LUAD) tumor identity, GFAP-positive staining suggesting glioblastoma multiforme, and Dicer1 mutation revealed by sequencing suggesting pleuropulmonary blastoma or sarcoma tumor origin. However, the tumor methylation profile pointed definitely toward LUAD. Molecular technologies such as next-generation sequencing and DNA methylation profiling have emerged as powerful adjunctive diagnostic tools for identifying and characterizing metastatic brain tumors. Large-scale studies have developed classifiers, such as the three-step DNA methylation BrainMETH classification system, that can differentiate metastatic brain lesions (from breast, lung, and melanoma primaries) from primary brain tumors, determine the tissue of origin, and even stratify breast cancer brain metastases by therapeutically relevant subtypes. This case of a rare metastatic GFAP-positive tumor with likely origin from the lung highlights the complexity of using these molecular technologies as diagnostic tools and reinforces the importance of using a combined approach using histological, immunohistochemical, and genetic techniques for future brain tumor diagnoses in order to avoid incorrect diagnosis based on overreliance on singular standard diagnostic approaches.
